# Drug‐Induced Immune Thrombocytopenia Secondary to Trimethoprim–Sulfamethoxazole

**DOI:** 10.1155/crdi/5522047

**Published:** 2026-02-03

**Authors:** Christopher Cimperman, Elysha Whitesel, Amy Ray, Nina-Naeger Murphy, Sara Atyia, Ismini Kourouni, Corrilynn Hileman

**Affiliations:** ^1^ Department of Medicine, Division of Palliative Care, MetroHealth Medical Center, Cleveland, Ohio, USA, metrohealth.org; ^2^ Case Western Reserve University School of Medicine, Cleveland, Ohio, USA, case.edu; ^3^ Department of Medicine, Internal Medicine Residency Program, MetroHealth Medical Center, Cleveland, Ohio, USA, metrohealth.org; ^4^ Department of Medicine, Division of Infectious Disease, MetroHealth Medical Center, Cleveland, Ohio, USA, metrohealth.org; ^5^ Department of Pharmacy, MetroHealth Medical Center, Cleveland, Ohio, USA, metrohealth.org; ^6^ Department of Medicine, Division of Pulmonary, Critical Care and Sleep Medicine, MetroHealth Medical Center, Cleveland, Ohio, USA, metrohealth.org

## Abstract

We report the case of a 55‐year‐old male with AIDS who developed severe thrombocytopenia following initiation of trimethoprim–sulfamethoxazole. Despite drug discontinuation and supportive measures, thrombocytopenia persisted, leading to the patient’s death. This case highlights the complexities of drug‐induced immune thrombocytopenia in immunocompromised patients and emphasizes the risks associated with trimethoprim–sulfamethoxazole.

## 1. Background

Drug‐induced immune thrombocytopenia (DIIT) causes platelet destruction, with resultant thrombocytopenia from drug‐dependent antibodies against platelets [1]. Overall, the risk of developing DIIT is rare. Although the risk of developing DIIT is low, certain medications are associated with higher rates of DIIT. Some examples include carbamazepine, eptifibatide, quinine, vancomycin, sulfamethoxazole, and trimethoprim. Typically, patients present with symptoms such as mucosal bleeding, petechiae, purpura, and an acute, severe drop in their platelet count (< 20,000 K/μL) within 5–10 days following the initiation of the offending medication [2]. We present a case of an immunocompromised patient who developed severe thrombocytopenia temporally related to the initiation of trimethoprim–sulfamethoxazole (TMP–SMX) during hospitalization.

## 2. Objective

To emphasize the potential risks associated with a commonly used antibiotic, TMP–SMX.

## 3. Case Report

A 55‐year‐old male with a medical history of HIV and polysubstance use disorder presented to the hospital following a syncopal episode. He reported that he had been eating less over the past few months, leading to a significant weight loss of 15–20 pounds. Physical examination was notable for cachexia, a noticeable cephalohematoma on the occiput, and left lower quadrant tenderness. Trauma workup prompted further imaging. A computed tomography scan of the chest, abdomen, and pelvis revealed extensive mesenteric and retroperitoneal lymphadenopathy.

The patient’s plasma HIV‐1 RNA level was 1.2 million copies/mL and CD4 count was 22 cells/mm^3^. He acknowledged a prolonged nonadherence to antiretroviral therapy (ART). In response, the patient was started on fixed dose bictegravir/emtricitabine/tenofovir alefenamide and TMP–SMX for *Pneumocystis jirovecii* pneumonia prophylaxis. Concurrently, an extensive workup was initiated to determine the cause of his lymphadenopathy. The differential diagnosis considered the possibility of AIDS‐associated malignancy with or without concurrent opportunistic infection. Although a lymph node biopsy was strongly recommended, the patient declined to undergo any procedures on multiple occasions.

Nine days into the patient’s hospital course, the development of moderate thrombocytopenia was noted with platelet counts decreasing from 346 K/μL to 58 K/μL over 3 days. The count further decreased to 8 K/μL the next day, accompanied by epistaxis and oral mucosal bleeding. Given the abrupt trajectory of this critical thrombocytopenia, an immune‐mediated mechanism was suspected. A peripheral blood smear did not show schistocytes, which made microangiopathic hemolytic anemia/thrombotic microangiopathy unlikely. Furthermore, LDH, haptoglobin, and reticulocyte counts were within normal limits, confirming the absence of hemolysis. The patient had not received any blood products, thus ruling out post‐transfusion purpura. The 4T score was three, making the probability of heparin‐induced thrombocytopenia low [3]. DIIT secondary to TMP–SMX was considered likely as the drug was started 6 days prior to the development of the thrombocytopenia. TMP–SMX was discontinued, antibody blood testing was sent to an external laboratory, and the patient was treated with intravenous immune globulin (IVIG) 1 g/kg for 2 days.

The patient’s platelet count remained low with the development of melena, encephalopathy, and hemorrhagic shock. In response, multiple platelet transfusions were initiated, as well as intravenous dexamethasone 40 mg daily for 4 days. A bone marrow biopsy was attempted, but the patient became agitated and uncooperative, leading to the procedure being aborted. Soon after, he required intubation for respiratory failure and airway protection in the setting of new severe lactic acidosis and distended abdomen upon examination. Computed tomography showed a closed loop small bowel obstruction (SBO) with pneumatosis intestinalis, concerning for small bowel ischemia.

Given his critical condition, the patient was deemed ineligible for surgical intervention. Further treatment was contrary to the patient’s expressed wishes as communicated by the next of kin. Consequently, the patient was transitioned to comfort care and died. Days following the patient’s death, the presence of TMP–SMX‐dependent platelet‐reactive antibodies by flow cytometric detection was confirmed (Versiti, Milwaukee, WI, USA), supporting the diagnosis of DIIT [4]. Additionally, two mycobacterial blood cultures returned positive for *Mycobacterium avium* identified by matrix‐assisted laser desorption/ionization‐time of flight (MALDI‐TOF) at the Ohio Department of Health, confirming disseminated *Mycobacterium avium* complex (MAC).

## 4. Discussion

DIIT is a rare yet significant complication associated with various medications, including antibiotics like TMP–SMX. This case report details a unique instance of a patient with AIDS who developed TMP–SMX‐induced immune thrombocytopenia that was a substrate to his death.

Thrombocytopenia, defined as a platelet count below 150,000 K/μL, is a well‐documented complication of HIV infection with a prevalence of 17.9% [5]. Thrombocytopenia can originate from various causes, reflecting the complex interplay of HIV infection, immune dysfunction, opportunistic infections, and adverse effects of treatment, including ART.

In this instance, the patient developed severe thrombocytopenia shortly after initiating TMP–SMX, which was indicated for opportunistic infection prophylaxis. TMP–SMX is commonly prescribed as prophylaxis against *Pneumocystis jirovecii* pneumonia and toxoplasmosis in immunocompromised patients, and while TMP–SMX is generally well tolerated, immune‐mediated adverse reactions such as thrombocytopenia can occur. One study estimated that 38 persons per million per week of exposure to TMP–SMX develop thrombocytopenic purpura, which was defined and diagnosed by a panel of hematologists [6]. The pathophysiology of TMP–SMX‐induced immune thrombocytopenia involves the formation of drug‐dependent antibodies that bind to platelet antigens, leading to accelerated platelet destruction [2]. This immune‐mediated process typically manifests within days to weeks after drug exposure and can result in severe bleeding complications.

Flow cytometric detection was used to confirm the presence of TMP–SMX‐dependent platelet‐reactive antibodies. This assay involves testing the patient’s serum or plasma against normal platelets, both with and without the suspected drug. A positive result occurs when the patient’s sample interacts with normal platelets only in the presence of the drug. Unfortunately, this test is not routinely performed due to it not being readily available at most institutions, has low sensitivity, and involves inherent complexities such as the poor solubility of the tested drugs in the aqueous medium [4].

Myelosuppression is another reason for thrombocytopenia that may occur as a result of antibiotic and other medication use. An example of this phenomenon is the reversible myelosuppression associated with linezolid. While linezolid can suppress all blood cell lines, thrombocytopenia is the most common and is dependent on duration of treatment greater than or equal to 2 weeks. The mechanism of this is unknown as there is no evidence of an immune‐mediated reaction [7].

In this case, TMP–SMX was discontinued the same day the platelet count decreased, as DIIT was clinically highly suspected. However, despite prompt removal of the offending medication, the platelet count did not begin to rise. To expedite recovery, IVIG and high‐dose glucocorticoids were initiated, but the thrombocytopenia remained refractory. Typically, the effects of IVIG and glucocorticoids are seen within 1–3 days and 2–14 days, respectively [8]. The exact mechanism by which glucocorticoids increase the platelet count is uncertain, but it is postulated that they may promote the apoptotic death of autoantibody‐producing lymphocytes and downregulate macrophage activity, which reduces platelet phagocytosis [9]. Similarly, IVIG is thought to decrease platelet destruction by inhibiting macrophage uptake of autoantibody‐coated platelets [10].

Given that our patient showed no signs of platelet recovery, it is possible that the patient’s concurrent disseminated MAC led to bone marrow suppression, contributing to the lack of response to treatment. Additionally, disseminated MAC may have contributed to his SBO, a complication that has been rarely cited, with only two previous case reports published to our knowledge [11, 12]. The pathophysiology likely results from infection through environmental exposure of the gastrointestinal tract, leading to localized MAC‐induced inflammation, that progresses to SBO [11].

### 4.1. Limitations

One limitation of this case report is the inability to definitively prove causation between disseminated MAC and the refractory nature of the thrombocytopenia due to the absence of a bone marrow biopsy or autopsy demonstrating bone marrow involvement. Another limitation of this case report is the lack of evidence that the SBO was due to disseminated MAC, though the SBO was largely unexpected.

## 5. Conclusion

The implications of this case underscore the importance of vigilant monitoring for early signs of adverse drug reactions and maintaining a high index of suspicion for DIIT in the context of unexplained thrombocytopenia. Timely identification and prompt intervention are crucial to optimizing patient outcomes and mitigating complications associated with thrombocytopenia; however, in some cases, as we have described, removal of the offending agent and therapies directed at immune‐mediated destruction may not be enough.

## Funding

No funding was received for this manuscript.

## Conflicts of Interest

The authors declare no conflicts of interest.

## Data Availability

The data that support the findings of this study are openly available in PubMed at https://pubmed.ncbi.nlm.nih.gov/.
